# A systematic review on the triggers and clinical features of type 2 myocardial infarction

**DOI:** 10.1002/clc.23230

**Published:** 2019-07-11

**Authors:** Guangqiang Wang, Na Zhao, Shu Zhong, Jianping Li

**Affiliations:** ^1^ Department of Cardiology The affiliated Yantai Yuhuangding Hospital of Qingdao University Yantai Shandong China; ^2^ Department of Rheumatology The affiliated Yantai Yuhuangding Hospital of Qingdao University Yantai Shandong China

**Keywords:** clinical characteristics, systematic review, trigger, type 2 myocardial infarction

## Abstract

Little is known about the correlation between triggering factors, clinical characteristics, diagnosis, and prognosis of patients with type 2 myocardial infarction (T2MI). The triggers and features of T2MI are linked to its diagnosis and prognosis. The Preferred Reporting Items for Systematic Reviews and Meta‐Analyses (PRISMA) guidelines were followed. A structured search of three databases (PubMed, Embase, and Medline) was undertaken to identify peer‐reviewed articles related to the triggers and clinical features of T2MI published between January 2012 and August 2018. Seven retrospective cohort studies and seven prospective cohort studies involving 3867 patients with T2MI were included. All selected studies were rated as being of high or acceptable quality. Nine studies revealed that the leading trigger of T2MI was arrhythmia, especially tachyarrhythmia. Six studies revealed that the proportion of single‐trigger T2MIs was higher than that of multiple triggers and two studies showed that two‐trigger cases formed the majority of multiple trigger cases. All included studies found that a greater prevalence of T2MI in the older population. Thirteen studies revealed that the patients with T2MI often had a previous relevant medical history. The leading trigger of T2MI is arrhythmia, especially tachyarrhythmia, and the majority of cases arise from a single trigger. Two‐trigger is the most common form of multiple‐trigger T2MI, which often occurs in older patients with cardiovascular risk factors or comorbidities. Non‐cardiovascular causes may be the triggering factors and are strongly associated with the diagnosis, treatment, and prognosis of T2MI.

## 1 INTRODUCTION

In 2007, the Global Myocardial Infarction (MI) Task Force released an expert consensus document classifying MI into five different subtypes.^1^ In 2012, the third universal definition of myocardial infarction provided a new classification of MI, which was based on etiology.^2^ In 2018, the fourth universal definition of MI brought forward a detailed classification of MI. Type 2 myocardial infarction (T2MI) is defined as MI not caused by plaque rupture, ulceration, erosion, or dissection with thrombotic obstruction, but secondary to myocardial oxygen supply‐demand imbalance related to an underlying cause. The definition of T2MI contains three aspects: (a) detection of a rise and/or fall in cardiac troponin (cTn) values with at least one value above the 99th percentile upper reference limit (URL); (b) clinical presentation of acute myocardial ischemia; and (c) evidence of an imbalance between myocardial oxygen supply and demand.^3^ T2MI may be multifactorial and caused by various conditions, such as anemia, arrhythmia, sepsis, infection, heart failure, respiratory failure, coronary artery spasm, hypertension, hypotension, aortic dissection, severe aortic valve disease, hypertrophic cardiomyopathy, or postoperative factors, but no specific criteria for the diagnosis of T2MI have been established.^4^ The lack of objective criteria for T2MI creates a diagnostic uncertainty, which has led to the inconsistent adoption of the classification in clinical practice. Thus far, the classification of T2MI is contentious because of the underlying pathological mechanisms and is based on expert consensus rather than evidence from prospective randomized controlled clinical trials. Patients diagnosed with T2MI are heterogeneous and have myocardial ischemia secondary to a variety of acute medical or surgical conditions.

Notably, little is still known about the correlation between triggering factors, clinical characteristics, diagnosis, and prognosis of patients with T2MI. Based on the current criteria, a diagnosis of T2MI could be applied to patients without atherosclerotic plaque disruption.^5^ Although patients with T2MI have higher rates of all‐cause death compared with those with type 1 myocardial infarction (T1MI), few studies report that the causes of T2MI are associated with a higher mortality rate. In addition, distinguishing different etiologies is essential for clinical management in patients with T2MI, mainly because a large spectrum of underlying causes for T2MI leads to an array of different treatment strategies.^6^, ^7^, ^8^, ^9^ Hence, the aim of this review is to assess the triggers of T2MI and to summarize the clinical features of patients with T2MI described in the literature.

## 2 METHODS

There is currently no consensus on the correct tool or measurement parameters to define T2MI, with different troponin I and T value ranges identifying T2MI currently in use. Moreover, there is no universal consensus on the cTn cut‐off points that clearly distinguish T2MI from MI. We therefore elected to include papers that had used any of the most commonly accepted metrics, according to a rise and/or fall in cTn values with at least one value above the 99th percentile URL.^3^

We followed the Preferred Reporting Items for Systematic Reviews and Meta‐Analysis (PRISMA) guidelines for this systematic review.^10^ The objective of this review was to identify all articles published in PubMed, Embase, and Medline databases between January 2012 and August 2018, which assessed the triggers of T2MI. Multiple variations of the following terms were utilized: cause, contributing factor, trigger, alternate factor, instigating factor, type 2 myocardial infarction, type II myocardial infarction, and myocardial infarction type 2. Additional references were identified from conference proceedings and/or citations in relevant review articles and assessed to find all available papers.

The inclusion criteria were as follows: (a) the definition, different causes, and baseline features of T2MI were mentioned; (b) an available clinical database was used; and (c) the study design met the requirement of high or acceptable quality assessment; especially, the single‐ and multiple‐trigger T2MI should be both included in selected studies. The exclusion criteria were as follows: (a) duplicate reporting; (b) lack of outcome data; and (c) non‐English publication.

Two investigators (G.Q.W. and N.Z.) independently evaluated the titles and abstracts of the articles retrieved using the search strategy. Abstracts that did not meet the inclusion criteria or met exclusion criteria were discarded. We selected the remaining studies for full‐text evaluation and data extraction. Any disagreements regarding the inclusion or exclusion of a study were solved by consensus, and, if doubt persisted, a third reviewer (J.P.L.) evaluated the reference. Therefore, we extracted data on the different causes of T2MI, which contained arrhythmia, anemia, respiratory failure, hypotension, infection and sepsis, heart failure, hypertension, postoperative factors, and other factors. Additionally, the data of multi‐trigger T2MI was extracted from selected studies.

## 3 RESULTS

**Figure 1:**
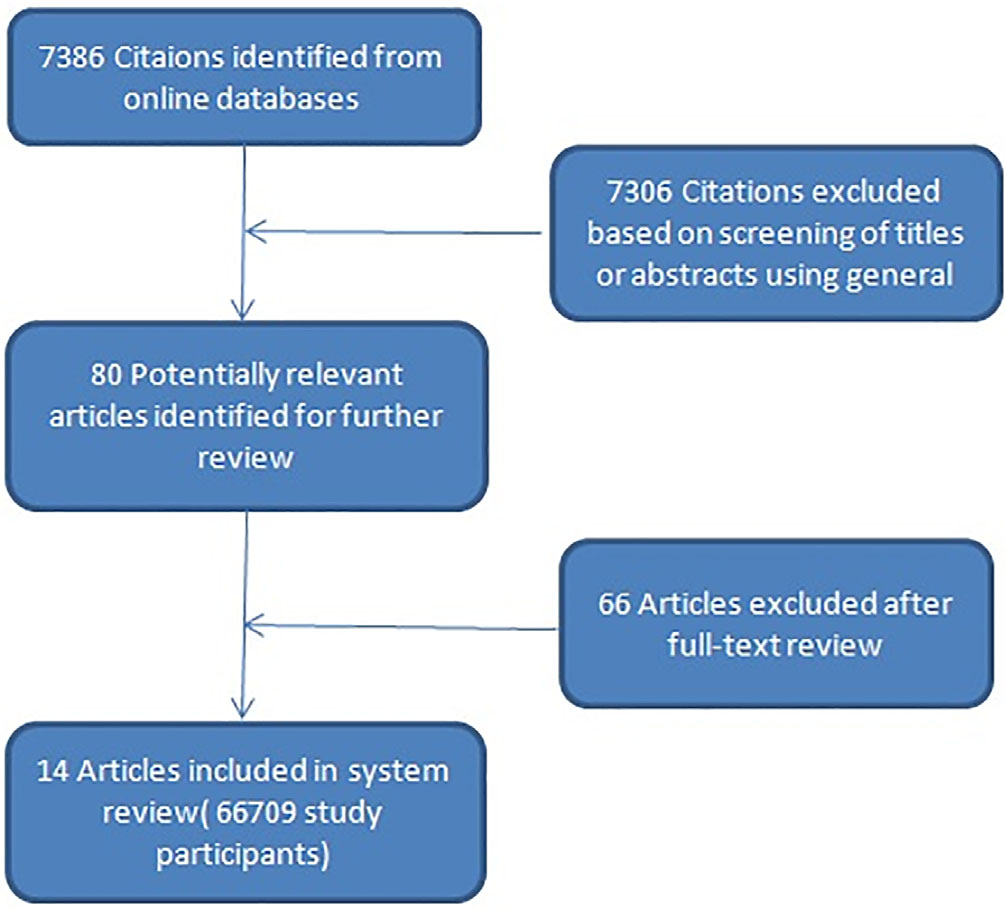
Flow diagram of the study selection process

Our literature search identified 7386 articles corresponding to the key terms; 7306 were excluded because they were not relevant based on the title and abstract. The full text of 80 articles was evaluated and 14 articles were ultimately included in this review. After screening the reference list of all included articles, no articles were added to this review. A detailed description of the selection process is presented in Figure 1. The 14 selected articles included 7 retrospective cohort studies and 7 prospective cohort studies involving 3867 patients with T2MI. The clinical characteristics of selected patients are shown in Table 1.

**Table 1:** Baseline characteristics and treatment of patients with T2MI

Characteristics	EI‐Haddad H 2012	Saaby L 2013	Baron T 2014	Saaby L 2014	Stein GY 2014	Szymariski FM 2014	Sandoval Y 2015	Lopez‐Cuenca A 2016	Landes U 2016	Smilowitz NR 2017	Radovanovic D 2017	Sandoval Y 2017	Nestelberger T 2017	Arora S 2018
Demographic/physical findings														
Age ±mean (SD)	67.2(21‐99)^b^	75±11	75.9±11.4	75±11	75±12	67.3 ± 13.2	61±8	72±12	74±10.4	74.3±12.2	70.8±13.3	60±15	72(58‐81)^b^	73(64‐81)^b^
Female sex (%)	126(42.7)	68(47.2)	657(46.8)	56(47.1)	55(43.4)	35(60.3)	13(59.0)	56(47.9)	38(35.5)	2(1.4)	387(35.5)	60(43)	92(38.3)	137(51.9)
Body mass index [kg/m^2^]±mean (SD)	NA	NA	NA	NA	25.8±4	27.56 ± 4.48	NA	NA	NA	NA	NA	29±8	26±2	26(23‐31)^b^
Systolic BP [mm Hg]±mean (SD)	NA	139±30.5	140.4±31.2	139±28.5	143±33	135.64 ± 23.17	142.5±18.5	135 ± 31	NA	NA	136±29	154±39	NA	NA
Diastolic BP [mm Hg]±mean (SD)	NA	77±17	79.3±18.0	NA	79±19	78.11±13.54	NA	72 ± 17	NA	NA	77±18	92±27	NA	NA
Heart rate [bpm]±mean (SD)	NA	113±20.5	97.8±30.5	112±20	95±25	81.39 ± 23.63	115.5±12.5	102 ± 36	NA	NA	84±25	100±30	NA	NA
NSTEMI(%)	NA	139(96.6)	1267(90.3)	115(96.6)	89(70.1)	42(72.4)	NA	116(99.1)	96(89.7)	NA	878(80.5)	109(77.9)	234(97.5)	NA
Medical history														
Hypertension(%)	NA	81(56.3)	760(54.2)	66(55.5)	108(84.9)	42(72.4)	14(64)	103(88)	87(81.3)	128(87.7)	766(73.5)	104(74.3)	182(75.8)	225(86)
Coronary artery disease (%)	NA	NA	NA	NA	50(39.7)	NA	NA	59(50.4)	68 (63.6)	NA	392(36.8)	24(17.1)	86(77.0)	NA
Previous myocardial infarction(%)	NA	39(27.1)	563(40.1)	26(21.9)	56(44.4)	15(25.9)	3(14)	19(16.2)	NA	28(19.2)	NA	15(10.7)	58(24.2)	NA
Previous coronary artery bypass surgery(%)	NA	14(9.7)	206(14.7)	9(7.6)	18(14.2)	NA	1(5)	12(10.3)	NA	27(18.5)	NA	NA	NA	51(19)
Previous percutaneous coronary intervention(%)	NA	25(17.4)	244(17.4)	15(12.6)	47(37.1)	NA	3(14)	40(34.2)	49 (46.2)	25(17.1)	NA	NA	64(26.7)	56(21)
Diabetes(%)	NA	40(27.9)	376(26.8)	28(23.5)	61(48)	14(24.1)	9(41)	52(44.4)	54(50.5)	58(39.7)	276(26.2)	43(30.7)	209(87.1)	110(42)
Dyslipidaemia(%)	NA	60(41.8)	NA	48(40.3)	93(73.2)	23(39.7)	9(41)	89(76.1)	82 (76.6)	102(69.9)	565(57.8)	61(43.6)	124(51.7)	131(50)
Obesity (BMI> 30) (%)	NA	NA	NA	NA	NA	NA	NA	NA	NA	NA	213(22.6)	NA	NA	NA
Cerebrovascular disease(%)	NA	31(21.5)	195(13.9)	24(20.2)	22(17.3)	2(3.4)	2(9)	20(17.1)	NA	NA	83(7.7)	18(12.9)	18(7.5)	NA
Peripheral vascular disease(%)	NA	18(12.5)	NA	17(14.3)	22(17.3)	9(15.5)	1(5)	11(9.4)	NA	11(7.5)	101(9.4)	3(2.1)	22(9.2)	46(18)
Arrhythmias(%)	NA	34(23.6)	394(28.1)	25(21.0)	18(14.2)	19(32.8)	1(5)	51(43.6)	NA	NA	170(15.6)	16(11.4)	NA	NA
Moderate‐to‐severe renal disease(%)	NA	20(13.9)	NA	NA	45(35.7)	19(32.8)	9(41)	NA	29(27.1)	48(32.8)	156(14.5)	32(22.9)	NA	66(25)
Peptic ulcers(%)	NA	NA	NA	NA	NA	7(12.1)	NA	NA	NA	NA	NA	NA	NA	NA
Thyroid disease(%)	NA	NA	NA	NA	NA	10(17.2)	NA	NA	NA	NA	NA	NA	NA	NA
Anemia(%)	NA	NA	NA	NA	NA	NA	NA	NA	NA	NA	331(33.5)	NA	NA	NA
Severe anemia(%)	NA	NA	NA	NA	NA	NA	NA	NA	NA	NA	146(14.8)	NA	NA	NA
Chronic obstructive pulmonary disease(%)	NA	36(25.0)	NA	31(26.1)	19(14.8)	NA	NA	17(14.5)	NA	32(21.9)	NA	NA	NA	NA
Heart failure(%)	NA	34(23.6)	288(20.5)	26(21.9)	32(25.6)	NA	4(18)	21(17.9)	NA	75(51.4)	73(6.8)	40(28.6)	NA	NA
Cancer disease(%)	NA	NA	NA	NA	NA	NA	NA	15(12.8)	4(3.7)	51(34.9)	93(8.7)	NA	NA	NA
Dementia(%)	NA	NA	NA	NA	NA	NA	NA	NA	4(3.7)	NA	37(3.4)	NA	NA	NA
Smoking(%)	NA	35(32.4)	765(54.5)	91(76.5)	20(15.8)	8(13.8)	NA	23(19.7)	44 (41.9)	96(65.8)	282(31.2)	52(37.1)	92(38.3)	80(30)
Family history (%)	NA	14(18.9)	NA	11(17.7)	NA	24(41.4)	NA	NA	NA	NA	243(29.5)	NA	29(12.1)	NA
Medicaments^a^														
Aspirin (%)	NA	NA	1041(74.2)	51(53.1)	NA	NA	NA	72(64.9)	NA	86(66.7)	983(90.7)	64(45.7)	NA	154(73)
P2Y12 inhibitors (%)	NA	NA	655(46.7)	13(13.5)	NA	NA	NA	46(41.4)	NA	33(25.6)	786(72.6)	6(4.3)	NA	72(34)
GP IIb/IIIa antagonist (%)	NA	NA	NA	NA	NA	NA	NA	NA	NA	11(8.5)	34(3.2)	NA	NA	NA
Heparin (%)	NA	NA	NA	NA	NA	NA	NA	NA	NA	NA	801(74.0)	NA	NA	NA
Beta‐blocker (%)	NA	NA	1146(81.7)	44(45.8)	NA	NA	NA	86(77.5)	NA	88(68.2)	595(55.4)	81(57.8)	NA	165(78)
ACEI/ARB antagonist (%)	NA	NA	937(66.8)	38(39.6)	NA	NA	NA	88(79.3)	NA	62(48.1)	566(52.4)	66(47.1)	NA	NA
Ca‐channel blocker (%)	NA	NA	NA	NA	NA	NA	NA	NA	NA	32(24.8)	161(15.1)	41(29.3)	NA	NA
Nitrate (%)	NA	NA	NA	NA	NA	NA	NA	NA	NA	NA	425(39.7)	NA	NA	NA
Aldosterone receptor antagonists(%)	NA	NA	NA	NA	NA	NA	NA	22(19.8)	NA	NA	NA	6(4.3)	NA	NA
Diuretic (%)	NA	NA	706(50.3)	NA	NA	NA	NA	70(63.1)	NA	NA	373(34.8)	46(32.8)	NA	NA
Statin (%)	NA	NA	926(66.0)	38(39.6)	NA	NA	NA	92(82.9)	NA	83(64.3)	698(64.8)	57(40.7)	NA	153(72)
Digoxin(%)	NA	NA	81(5.8)	NA	NA	NA	NA	NA	NA	NA	NA	6(4.3)	NA	NA
Oral anticoagulant(%)	NA	NA	219(15.6)	10(10.4)	NA	NA	NA	44(39.6)	NA	NA	NA	20(14.3)	NA	NA
Anti‐arrhythmic(s) (%)	NA	NA	NA	NA	NA	NA	NA	NA	NA	NA	NA	3(2.1)	NA	NA
Intervention														
Coronary angiography (%)	NA	31(21.5)	504(35.9)	28(25.5)	34(27)	NA	1(5)	46(39.3)	NA	19(13.0)	660(60.5)	13(9.3)	58(24.2)	68(26)
PCI (%)	NA	NA	175(12.5)	4(3.4)	17(13.5)	NA	0(0)	11(9.4)	NA	8(5.5)	557(51.1)	1(0.7)	7(2.9)	32(12)

Abbreviation IQR, interquartile range;PCI, percutaneous coronary intervention.

a Referred to patients alive at discharge.

b Stands for median (IQR).

To evaluate the quality of included studies, we applied the improved Newcastle‐Ottawa Quality Assessment Scale (NOS) for nonrandomized studies.^11^ All prospective or retrospective cohort studies received a rating of high (NOS ≥7) or acceptable quality (NOS ≥6) in this systematic review, respectively.

The baseline clinical characteristics and treatment of patients with T2MI are summarized in Table 1.^4^, ^12^, ^13^, ^14^, ^15^, ^16^, ^17^, ^18^, ^19^, ^20^, ^21^, ^22^, ^23^, ^24^ The mean age of patients with T2MI was over 60 years; most studies revealed that the percentage of male patients ranged from 52.8% to 98.6% in patients with T2MI, but the studies of Szymariski et al., Sandoval et al., and Arora et al. found percentages of female patients of 60.3%, 59%, and 51.9%, respectively.^17^, ^18^, ^24^ The ratio of patients with T2MI presenting with non‐ST segment elevation myocardial infarction (NSTEMI) ranged from 70.1% to 97.5% (mean value: 87.1%); all studies found that patients with T2MI often had previous relevant medical history, but EI‐Haddad et al. did not describe medical history findings.^12^ Only seven studies described the multiple medications used by patients with T2MI after discharge, which mainly included aspirin, P2Y12 inhibitors, beta blockers, angiotensin‐converting enzyme inhibitor (ACEI), angiotensin II receptor blocker (ARB), and statins. Detailed medication regimens are also described. Eleven studies showed 5% to 60.5% (mean value: 26.1%) of patients with T2MI were diagnosed by coronary angiography, and 10 studies showed that 0% to 51.1% (mean value: 11.1%) of patients with T2MI received percutaneous coronary intervention (PCI).

The triggers for T2MI are shown in Table 2. The common causes of T2MI were arrhythmia, anemia, hypertension, sepsis/infection, respiratory failure, hypotension, heart failure, and postoperative factors. Nine studies revealed the leading cause of T2MI was arrhythmia, especially tachyarrhythmia. On the contrary, Radovanovic et al. and Stein et al. found that the most common cause was anemia,^4^, ^16^ while EI‐Haddad et al., Szymariski et al., and Arora et al. revealed the most common triggers were postoperative factors, coronary artery spasm, and sepsis/infection, respectively.^12^, ^17^, ^24^

**Table 2:** Different triggers of T2MI in selected studies

		Instigating factors										
Study	Total	Arrhythmia	Tachyarrhythmia	Bradyarrhythmia	Anemia	Respiratory failure	Hypertension	Sepsis/infection	Hypotension	Heart failure	Postoperative	Others
EI‐Haddad H 2012	295	9	9	NA	31	31	32	71	NA	10	82	29
Saaby L 2013	144	46	42	4	30	30	1	9	NA	NA	NA	13
Baron T 2014	1403	331	331	NA	186	19	30	246	NA	260	NA	184
Saaby L 2014	119	48	44	4	21	38	NA	14	NA	NA	NA	14
Stein GY 2014	127	22	NA	NA	39	NA	NA	30	NA	14	18	18
Szymariski FM 2014	58	15	NA	NA	11	NA	9	NA	NA	NA	NA	23
Sandoval Y 2015	22	8	8	NA	NA	NA	6	NA	8	NA	NA	NA
Lopez‐Cuenca A 2016	117	43	43	NA	6	NA	NA	NA	NA	18	NA	NA
Landes U 2016	107	36	36	NA	NA	NA	NA	NA	NA	NA	27	NA
Smilowitz NR 2017	146	53	43	10	29	25	29	43	20	NA	NA	NA
Radovanovic D 2017	685	204	204	NA	331	NA	NA	36	36	NA	51	NA
Sandoval Y 2017	140	71	66	5	11	57	59	NA	34	NA	NA	7
Nestelberger T 2017	240	139	123	16	7	3	64	NA	NA	NA	NA	NA
Arora S 2018	264	16	NA	NA	19	47	19	74	7	28	8	46

**Figure 2:**
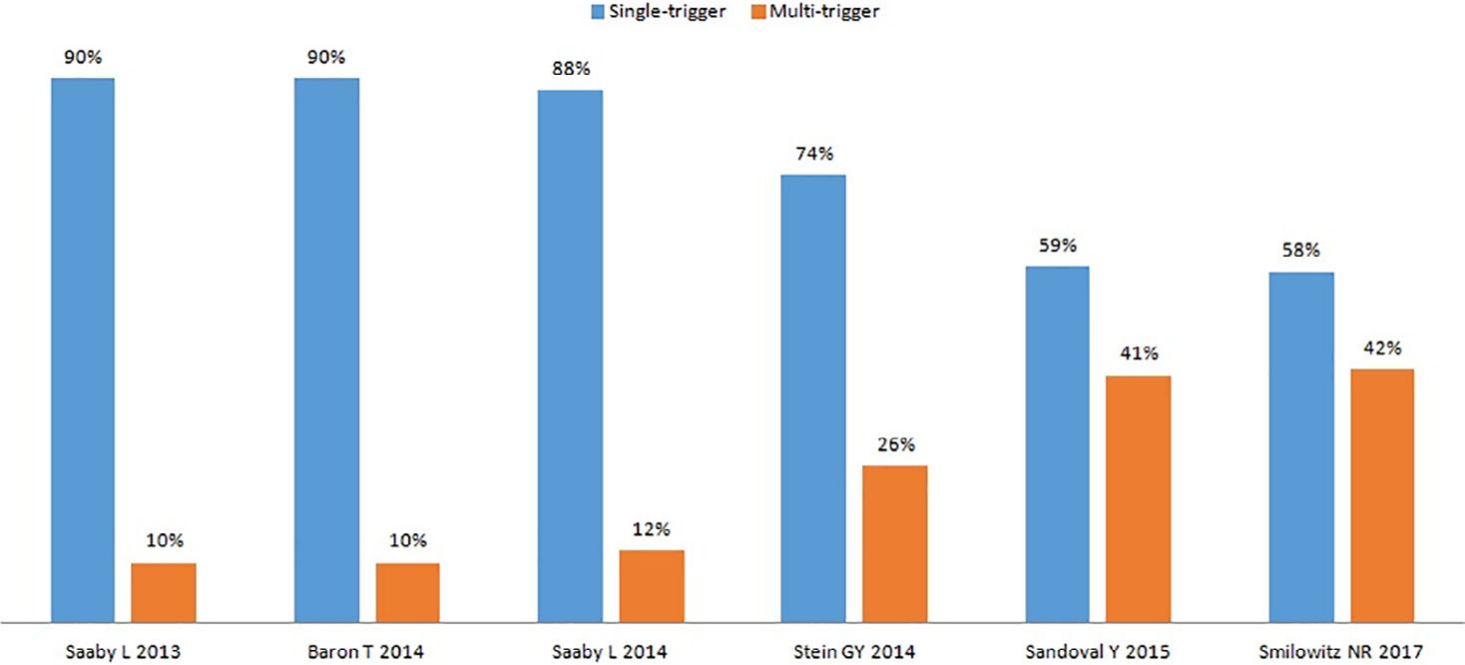
Column chart showing the comparison between single‐trigger and multi‐trigger T2MI in selected studies

**Figure 3:**
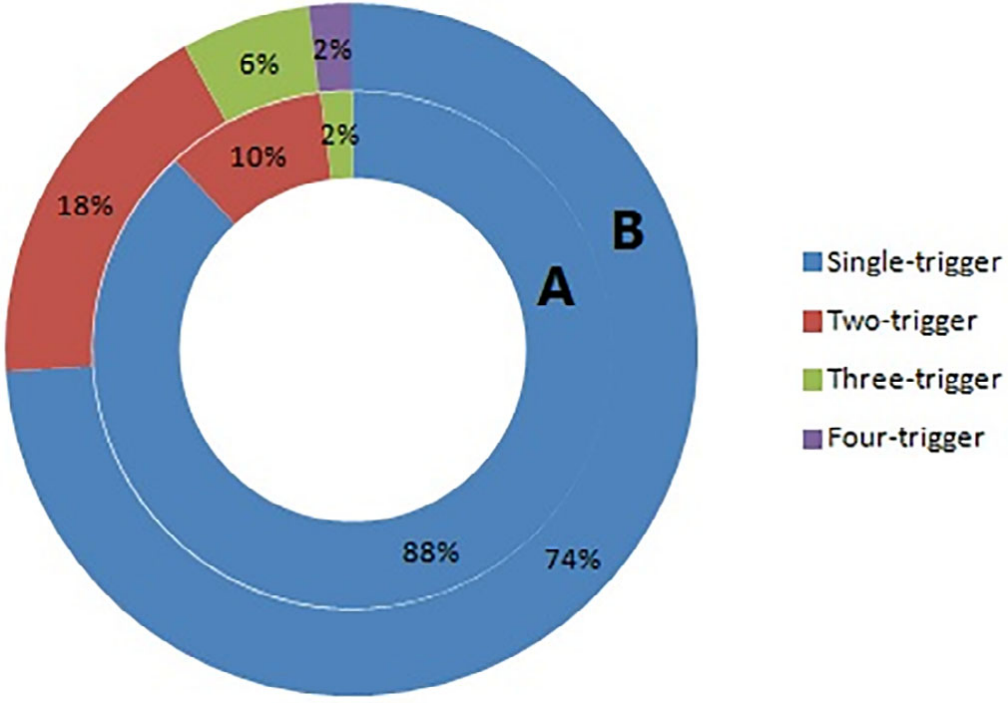
Doughnut chart showing the multi‐trigger T2MI frequencies in patients with T2MI. A, Saaby et al; B, Stein et al

Figure 2 shows the comparison between the single‐ and multiple‐trigger T2MI in six selected studies and shows that the proportion of single‐trigger T2MI was higher than that of multiple‐trigger T2MI, while Figure 3 shows that 2‐trigger T2MI constituted the majority of multiple‐trigger cases.^15^, ^16^

## 4 DISCUSSION

A key finding of this review is that the clinical characteristics of patients with T2MI, which were similar to those of found by Gupta et al.^25^ Patients with T2MI were older, more often men, more frequently presented with NSTEMI, and had a higher prevalence of cardiovascular risk factors or comorbidities, such as hypertension, smoking, dyslipidemia, diabetes, obesity, heart failure, impaired renal function, anemia, coronary artery disease, atrial fibrillation, cancer, peripheral artery disease, and chronic obstructive pulmonary disease. In this review, most selected studies revealed that the number of men with T2MI was higher than that of women, while a reduced number of studies found the opposite.^17^, ^18^, ^24^ For example, Gupta et al. described T2MI was more common in females compared with T1MI.^25^ Among the clinical characteristics of T2MI, we concluded that cardiovascular risk factors or comorbidities might be the most important causes of T2MI and affect the prognosis in these patients. The poor short‐ and long‐term prognosis is not entirely surprising since T2MI typically occurs among older patients with greater comorbidities and is identified in the context of hemodynamic instability.^26^ T2MI has a high mortality, and most deaths among patients with T2MI are due to noncardiovascular causes. Putot et al. revealed that T2MI was associated with a worse in‐hospital prognosis than T1MI resulting from non‐cardiovascular events.^27^ Smilowitz et al. showed that the rates of non‐cardiovascular deaths were 82.4%, 68.2%, and 64.4% in patients with T2MI during hospital admission, 30 days post‐discharge, and at intermediate‐term follow‐up, respectively.^21^ Lambrecht et al. showed T2MI led to a significantly higher long‐term mortality than T1MI, and noncardiovascular causes (57.4%) of death predominated in patients with T2MI, mainly including respiratory system diseases (20.6%), neoplasms (13.2%), and other noncardiovascular diseases (16.2%).^28^ Arora et al. found that 23 (50%) patients with T2MI died from infection or sepsis, which constitute noncardiovascular death causes.^24^ Few studies confirm that T2MI has worse outcomes independent of severe concomitant diseases. On the other hand, patients with T2MI were less likely to undergo coronary angiography (CAG) or PCI or to receive secondary preventive treatment than patients with T1MI. The impact of anti‐thrombotic and/or antiplatelet therapy, as well as the role of reperfusion in patients with T2MI due to mild atherosclerotic coronary stenosis might be beneficial or effective, but in patients without plaque rupture this benefit is uncertain and there might even be a detrimental effect or contraindication to treatment in many cases.^19^, ^29^ Otherwise, patients with T2MI often receive specific treatments for concomitant diseases, such as anticoagulants for atrial fibrillation or diuretics for heart failure. Regardless of the definition, we agree that the optimal medical therapy should be based on the cause of T2MI.

This review systematically evaluated T2MI triggers and listed the leading cause and other common causes, especially in single‐trigger cases; our findings also shed light on the underlying etiologies, which may help improve the decision regarding treatment options. In the TRACER trial, the most frequent potential provoking factor for T2MI was tachyarrhythmia (38.2%), which is consistent with our findings.^30^ A meta‐analysis also demonstrated that the most common associated arrhythmia was tachyarrhythmia, especially atrial fibrillation, in patients with T2MI.^25^ Most patients with T2MI died from noncardiovascular causes during long‐term follow‐up. However, few studies described the association between cardiovascular trigger and prognosis in patients with T2MI, which is crucial for clinicians. Therefore, opportunities exist to identify high cardiovascular risk patients and facilitate evidence‐based therapies geared toward improving their outcomes.^31^

Distinguishing patients with T2MI from those with T1MI might be particularly challenging and requires a careful clinical assessment. It is crucial that the differentiation is made whether the myocardial injury is likely to be due to plaque rupture or to an imbalance between myocardial oxygen supply and demand, because the management of these two conditions is quite different. While the treatment of T1MI primarily includes antithrombotic therapy and/or revascularization, as clinically appropriate, the management of T2MI is more varied because several different mechanisms may be responsible for the pathological ischemic imbalance. Although visualization of a culprit lesion on angiography is often required to define a T1MI by clinicians and/or researchers, it may result in misclassification. Among patients with T2MI, in which the index event appears to be related to underlying undiagnosed coronary artery disease, CAG should be considered. Therefore, it should be routinely used to differentiate T1MI and T2MI, but not confirm the diagnosis of T2MI. The diagnosis of T2MI should be done by combining the detection of acute myocardial injury, clinical presentation of acute myocardial ischemia, and evidence of myocardial oxygen supply‐demand mismatch. Sandoval et al. stressed that using objective evidence of myocardial ischemia to diagnose T2MI might result in a more precise and specific disease definition.^32^ Moreover, establishing the trigger factors is essential for the diagnosis of T2MI.

To date, T2MI is a controversial matter in both clinical practice and research trials. There was no standardized clarification of the diagnostic criteria for T2MI. Although various suggestions have been made, these have not been widely accepted. Most importantly, there is an urgent need for evidence‐based diagnostic and therapeutic strategies, primarily randomized, controlled clinical trials.^19^

There are several limitations to this systematic review. First, it is based on a relatively small number of selected patients. T2MI cases are not representative of the local population they were been derived from, and the study population is heterogeneous. The study period is narrow and might not be able to represent the whole spectrum of patients with T2MI adequately. In addition, selection bias may be present due to the heterogeneity of included patients due to the subjectivity of the diagnostic criteria for T2MI and to the different diagnostic methods used.^33^ Second, the objectives of individual studies are variable. This has potential consequences on the procedures used and on the reliability of how underlying conditions and their distribution among T2MI cases were established. Third, this review only includes retrospective and prospective cohort studies, but not prospective randomized controlled studies. Several selected studies have incomplete documentation for the key factors in this investigation. All individual studies lack information on how the underlying causes were established. Despite a rigorous statistical analysis, residual confounding and selection bias are likely to be present.

## 5 CONCLUSION

The leading trigger of T2MI was arrhythmia, especially tachyarrhythmia, and single‐trigger cases represented the majority of cases. Among multiple‐trigger cases of T2MI, two‐trigger cases are the most common. T2MI often occurs in older patients with cardiovascular risk factors or comorbidities. As for the triggering factors, non‐cardiovascular causes are closely related to the diagnosis and prognosis of T2MI. Furthermore, the optimal medical therapy should be decided based on the trigger of T2MI. Although recent data are promising, more prospective randomized controlled studies are necessary to verify the impact of different triggers on the diagnosis, treatment, and prognosis of T2MI.

## CONFLICT OF INTEREST

The authors declare no potential conflicts of interest.
